# Ten quick tips for fuzzy logic modeling of biomedical systems

**DOI:** 10.1371/journal.pcbi.1011700

**Published:** 2023-12-21

**Authors:** Davide Chicco, Simone Spolaor, Marco S. Nobile

**Affiliations:** 1 Institute of Health Policy Management and Evaluation, University of Toronto, Toronto, Ontario, Canada; 2 Dipartimento di Informatica Sistemistica e Comunicazione, Università di Milano-Bicocca, Milan, Italy; 3 Microsystems, Eindhoven University of Technology, Eindhoven, the Netherlands; 4 Department of Environmental Sciences Informatics and Statistics, Ca’ Foscari University of Venice, Venice, Italy; bioinformatics.ca, CANADA

## Abstract

Fuzzy logic is useful tool to describe and represent biological or medical scenarios, where often states and outcomes are not only completely true or completely false, but rather partially true or partially false. Despite its usefulness and spread, fuzzy logic modeling might easily be done in the wrong way, especially by beginners and unexperienced researchers, who might overlook some important aspects or might make common mistakes. Malpractices and pitfalls, in turn, can lead to wrong or overoptimistic, inflated results, with negative consequences to the biomedical research community trying to comprehend a particular phenomenon, or even to patients suffering from the investigated disease. To avoid common mistakes, we present here a list of quick tips for fuzzy logic modeling any biomedical scenario: some guidelines which should be taken into account by any fuzzy logic practitioner, including experts. We believe our best practices can have a strong impact in the scientific community, allowing researchers who follow them to obtain better, more reliable results and outcomes in biomedical contexts.

## Introduction

Fuzzy logic is a type of many-valued logic propositional calculus where there are more than two truth values and which can be employed to express the concept of partially true or partially false statuses. It is intended in contrast to Boolean logic, where an element can only be completely true or completely false.

Thanks to fuzzy logic’s capability to formally describe undefined contexts and scenarios, researchers and scientists often employed fuzzy logic models in medical and healthcare research studies [1–6], where the conditions of patients or the interpretation of a biological aspects often are not defined in a strictly Boolean way.

Fuzzy logic, for example, can be applied to data of laboratory test results of patients at risk of heart attack to predict the chance that they might experience a cardiovascular disease [7]. Multiple clinical features should be taken into account to predict a heart disease episode, and the relationship between their values and the outcome is not linear. In cases like this, fuzzy logic can generate reliable results.

Even if fuzzy logic models have become easy to use in biomedical research, it is also easy to make mistakes that can corrupt the final results and outcomes of a study, especially for beginners and unexperienced users. We therefore present here a short list of guidelines to keep in mind to use fuzzy logic correctly, based on our experiences and on what we learnt from the scientific literature.

**Fig 1:**
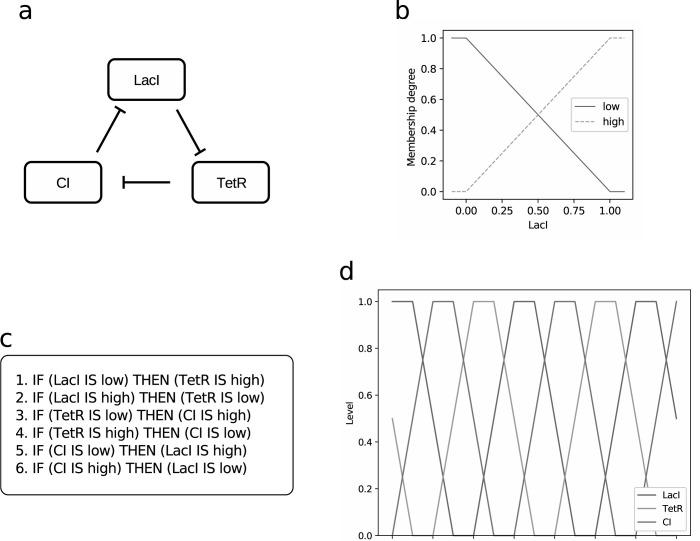
Example of a fuzzy logic-based model representing the repressilator, an oscillatory genetic regulatory network [10]. (a) Schematic representation of the variables present in the model and their interactions. (b) Fuzzy sets representing each of the variable’s states (only LacI is shown). (c) Fuzzy rule base of the model. (d) Resulting oscillatory dynamic of the fuzzy model. We partially adapted these figures from [15], where the original figures were released under the CC BY-NC 4.0 DEED (Creative Commons Attribution-NonCommercial 4.0 International) license.

The education collection of the *PLOS Computational Biology* journal published a list of quick tips for modeling high-resolution protein 3D structures [8] and a list of quick tips for dynamically modeling bioprocesses [9], but never released any guidelines on fuzzy logic modeling. We fill this gap by presenting the current study, where we introduce ten quick simple tips on how to design fuzzy logic models which can describe biological or health systems, by avoiding common mistakes and pitfalls. To facilitate the reading and understanding of our tips, we also introduce a toy fuzzy model of the repressilator [10], an oscillatory genetic regulatory network (Fig 1A).

For a complete overview and formal definitions of fuzzy models and their components, we refer the interested readers to general review and books regarding fuzzy logic and its applications [11–13]. However, in what follows we provide basic concepts and definitions that will be encountered in our ten quick tips.

### Fuzzy set

A fuzzy set represents an extension of the ordinary set [14], whose boundaries are not sharp. This property allows for elements to belong to a fuzzy set to a “certain degree,” varying continuously between 0 and 1, making it possible to represent concepts of human reasoning where it is not possible to define a clear threshold separating to different concepts. For example, two fuzzy sets can be used to define a smooth transition from “cold” to “warm” water in a continuous way, without a clear, crisp threshold specifying at which temperature the water stops being cold and starts being warm. Analogously, in biology, fuzzy sets can be used to represent gene expression (for example, highly expressed or lowly expressed) or the concentration of a molecule (high concentration, low concentration). In our repressilator model [15], for example, we represented protein concentration by two fuzzy sets (Fig 1B). Fuzzy sets embody a form of vagueness and uncertainty about some measurement, that is typical of human language, but that is not related to uncertainty derived by randomness, as fuzzy sets do not represent probability distributions [14].

### Linguistic variable

Fuzzy sets can be used to define linguistic variables, that is, variables that can assume as values both words in some natural or artificial language, and numbers [16]. Linguistic variables connect the quantitative world of numbers with the qualitative world of words thanks to fuzzy sets. To define a linguistic variable, one needs to define a universe of discourse, that is an ordered collection of numerical values that the variable can assume, and partition it with fuzzy sets. Each fuzzy set is then associated to a linguistic term, that is, the linguistic value that the linguistic variable can assume. For example, referring again to gene expression, the linguistic variable “expression of gene A” can be represented by partitioning the universe of discourse into two fuzzy sets, with the linguistic terms “low expression” and “high expression.”

### Fuzzy rule

In classical logic, IF/THEN rules are used to express implication and represent “pieces of knowledge.” These rules can be extended into fuzzy IF/THEN rules [17], logic rules in which the antecedent (the part before the THEN), or both antecedent and consequent (the part after the THEN), are fuzzy sets rather than crisp. Fuzzy rules generally appear in the following form:

IFXISaTHENYISb, (1)

where *X* and *Y* are two linguistic variables, *a* is a linguistic term of *X* and *b* is either a linguistic term of *Y*, or a function of the variables appearing in the antecedent. The antecedent of a fuzzy rule can also include logic operators, such as AND, OR, NOT. The peculiarity of fuzzy rules is that, given some input values of the variables appearing in the antecedent, they can be satisfied to a certain degree, since they employ fuzzy sets. The degree of satisfaction of a rule is defined as the degree to which the given values of the input linguistic variables match the antecedent of the rule. We provide an example of fuzzy rule based in Fig 1C: these rules represent the negative interactions existing between the three variable of our repressilator model [15].

### Fuzzy inference system

Linguistic variables and fuzzy rules can be used to define a fuzzy inference system [12]. Fuzzy inference is the process of mapping a given input to an output by means of fuzzy logic. Fuzzy inference generally consists of the following steps:

1. Fuzzification of crisp inputs into their linguistic variables and evaluation of their membership degree.
2. Rule evaluations.
3. Aggregation of rules’ outputs into a single output.
4. Defuzzification (optional) of the fuzzy rules’ output into a single crisp value.

Albeit sharing the general steps described above, there are several fuzzy inference methods in the literature that differ in the definition of the consequents of the fuzzy rules and how they aggregate the rules evaluations. The two most widespread and adopted methods are the Mamdani [18] and the Sugeno [19] inference methods. We refer the interested readers to their respective references for detailed explanations on their working. Worthy of note, fuzzy inference systems can be “connected,” in order to feed the output of one system as input to a downstream one, creating a fuzzy inference network [20]. Such networks can be depicted as a graph, where nodes represent linguistic variables, and arcs the presence of some fuzzy rules governing them. These networks can be defined with arbitrary connections, including cycles and feedback loops, a feature that proves particularly useful to model the interactions existing in biological systems (for example, [21–23]).

Our best practices are intended for beginners and students, but should be kept in mind by experts, too.

## Tip 1: Keep in mind that all models are wrong but some of them are useful, even in fuzzy logic

When facing a complex biomedical scenario, involving multiple factors and complex environments, one might feel discouraged and think that no good mathematical model would be able to precisely represent it. For those moments, we would like to recall that the famous quote by George E. P. Box (“All models are wrong, but some are useful”) is true for fuzzy logic, too: even if not perfect, a fuzzy logic model describing a particular biological or medical scenario can be an advantageous and handy tool to formally describe and study the scenario itself.

Of course, the initial fuzzy logic model can be improved, modified, and corrected when new information becomes available or when the scenario understanding increases. However, it is important to understand that, even for complex environments, a simple fuzzy logic model can always be of help. As Sheryl Sandberg says: “Done is better than perfect.”

For example, considering our repressilator model [15] in Fig 1, it could be argued that this model avoids taking into account the role of transcriptional and translational machinery in the cell, or depletion mechanisms for the transcription factors. However, this simple model is sufficient to display a stable oscillatory behavior, as shown in the dynamics in Fig 1D, which is the characteristic behavior of this gene network.

So, here is the core of this first tip: do not get discouraged if the biomedical scenario you would like to describe through fuzzy logic is complex. No scenario is too complex for fuzzy logic modeling. Start with a simple model, and then improve it until you represent all the elements involved (Tip 2).

## Tip 2: Start small and simple, add complexity only if necessary, and avoid transitive closures

Once you decided it is worth trying to describe a biomedical scenario with a fuzzy logic model, you might wonder how to start. We have a clear piece of advice for you: as recommended for machine learning methods selection [24], we suggest you to start with the simplest model possible. You can start by including just a few variables, interactions, statuses, and sets in your fuzzy logic model, test the model, double-check its results, and then add new elements, and repeat the cycle. Of course, you need to begin with the most important interactions and traits of the model, and then add the less relevant aspects later on.

Do not start by including all possible the variables, statuses, interactions, and fuzzy sets from the beginning. If a more complex version of your fuzzy model stops working, get back to the previous, easier version, and stick with it. Your fuzzy logic model does not need to be particularly complicated: the simpler your model is, the easier will be to keep under control and to debug.

In the machine learning community, some researchers sometimes employ complex computational models just to impress the readership or the audience. We suggest to do the opposite: stay simple. Leonardo Da Vinci used to say: “Simplicity is the ultimate sophistication.” It is always true, even for fuzzy logic.

Especially when modeling intricate networks of interactions, such as biological systems, you might be tempted to add all known interactions to your model. However, you should keep in mind that every interaction (or fuzzy rule) in your model should represent a known causal link between two variables, and not just a relationship arising from correlation, to avoid accounting multiple times for the same phenomenon in your model.

Our repressilator model [15] comprises of three linguistic variables, corresponding to 3 transcriptional factors named LacI, TetR, and CI. Each of these three proteins exert a feedback inhibition on the next protein in the loop (Fig 1A). For example, LacI inhibits TetR, which in turn inhibits CI, thus the fuzzy rules should represent these interactions separately (Fig 1C), avoiding shortcuts such as “LacI induces CI.”

## Tip 3: Keep in mind that the best results for a scientific project arrive only if domain experts are involved

Once you identified a particular biological or healthcare scenario that you would like to model through fuzzy logic, you might feel tempted to study the biomedical context on your own and then to try to develop the fuzzy logic model on your own. In these cases, we suggest to change your plans: go and look for someone who knows the biomedical scenario you are trying to describe and propose them to collaborate with you on this task.

An effective modeling can happen, in fact, only when the fuzzy model researcher works together with an expert of the analyzed field: the feedback and information provided by the domain expert is invaluable and can definitely help creating a better fuzzy logic model. Therefore, if the scenario refers to molecular biology, go and look for a biologist in your scientific centre, university, company, or on the internet. If the scenario instead is about data of patients with myocardial infarction, try to get in touch with a cardiologist or a medical student, and propose them to help you with the model development. They will be able to guide you in the model development and to avoid common mistakes or confusion in your reasoning.

Biomedical informatics research of high impact can happen only when computational experts and biomedical experts meet and collaborate [25]: this is always true, also for fuzzy logic modeling.

As Pedro Domingos in his *few things* to know on machine learning [26]: “The organizations that make the most of machine learning are those that have in place an infrastructure that makes experimenting with many different learners, data sources, and learning problems easy and efficient, and where there is a close collaboration between machine learning experts and application domain ones.”

Of course, it is true fuzzy logic as well: so go out and look for collaborators having different scientific background from yours.

## Tip 4: Know your variables in detail and define one universe of discourse per linguistic variable

Identify the most important variables of your model: determine the actors, objects, or concepts that are fundamental to represent the phenomenon. Add to the model any other variable that you know is relevant, for example, those that regulate, or are regulate by, your initial set of variables.

You must spend some effort to properly characterize these variables. In particular, it is important to keep in mind that a linguistic variable has only 1 universe of discourse, which will be shared by all fuzzy sets. The universe of discourse can be finite (for example, a percentage), semi-finite (for example, a Kelvin temperature), or infinite depending on the variable to be modeled.

The degree of membership represented by a fuzzy set should be interpreted as a degree of similarity to a prototype condition [27] (for example, low fever or high fever). Thus, fuzzy sets belonging to the same variable should represent different possible states of the same quantity. In our repressilator model [15], for example, linguistic variables (Fig 1B) consist of two fuzzy sets, representing only “low” and “high” protein concentrations. The universe of discourse of these variables represent only a concentration, and not other quantities (for example, affinity for binding, activation state, etc.).

Defining a membership function for a complex phenomenon can sometimes be difficult, since we need to map every point of the universe of discourse to a value between 0 and 1. Try to characterize every universe of discourse with only one physical measure/property in order to make this process easier.

## Tip 5: Select the salient interactions, keep your rule base as small as possible, but cover all fuzzy sets with fuzzy rules

Add to the model only components and interactions that play a role in the biomedical system that you are investigating. As already mentioned in Tip 2, keeping your model small is important to maintain the interpretability and explanatory power of your model. This principle is valid also when considering the number of fuzzy rules in your model.

However, you should also make sure that all the fuzzy sets specified in your linguistic variables appear at least in one rule. Fuzzy sets that do not appear in any rule will not contribute to any inference, thus resulting redundant and not contributing to the calculation of the outputs of your model. If this happens, consider defining new fuzzy rules that cover these sets or remove the fuzzy sets (and the parts of the universe of discourse) that are not needed in your model.

For our repressilator model [15], we started adding two fuzzy sets per variable, and we covered all of them with at least one rule (Fig 1B and 1C). Two fuzzy sets per variable and six rules are enough to represent the characteristic behavior of the system, that is, stable oscillations.

## Tip 6: Choose the appropriate fuzzy reasoner for your scientific problem

Keep in mind that different inference methods exist in fuzzy logic, and each of them has its pros and cons. Thus, it is important to choose the one that best fits the needs of your research. Mamdani inference [18] is generally regarded as more straightforward and easier to understand for nonexperts, since it uses fuzzy sets in the consequents of the fuzzy rules. For the same reasons, Mamdani models are also regarded as interpretable and easy to inspect. However, Mamdani inference has a higher computational cost with respect to Sugeno inference [19]. Sugeno inference adopts functions in the consequents of the fuzzy rules, which makes fuzzy inference computationally efficient, at the cost of making models less easy to interpret and characterized by an increased number of free parameters. To overcome these last limitations, we suggest to define models employing 0- or first-order Sugeno inference, that is, employing a constant value or a linear functions in their consequents, respectively. Due to the possibility of using arbitrary functions in the consequent, Sugeno reasoning systems are usually employed for the modeling, simulation, and control of dynamic systems.

As soon as the linguistic variables, the membership functions, and the fuzzy rules are defined, and a proper fuzzy reasoner is selected, the system is ready to be implemented and tested. Among the available open-source libraries for fuzzy reasoning, the most straightforward to use is Simpful [15], a Python library that provides a simplified interface to build arbitrarily complex systems and supports both Sugeno and Mamdani reasoning. We provide more information about Simpful in Tip 9.

## Tip 7: Calibrate the system properly

Caveat emptor: we originally designed and wrote these tips for beginners, but this tip can probably be understood only by fuzzy logic experts.

Even though a modeler can have a full knowledge of the elements of the system—and convey in the fuzzy rules all mechanisms that govern their interactions and dynamic behavior—the result of the inference should still be compared with the available data in order to check that the fuzzy model properly reflects the experimental evidence. For example, if the rule reads “if gene transcription is high then protein concentration is high,” the experiments should confirm this mechanism. Whenever a modeler experiences any discrepancies between the fuzzy inference and the experimental data, there might be multiple possible motivations.

The first suspect are the membership functions of the linguistic variables. As a matter of fact, especially in life sciences, it is often the case that a quantitative characterization of “low” and “high” states for a given variable (for example, concentration, expression) are not immediately available. Hence, the parameters used to define the membership functions (as described in the Introduction) might be slightly offset for the system under investigation, leading to a strength of the fuzzy rules that does not reflect the expectations of the modeler which, in turn, affects the final output of the fuzzy inference. In this case, a domain expert should review all membership functions and tweak the parameters where needed, in order to maximize the adherence of the fuzzy sets to the actual prototype conditions.

Another possibility is that the parameters used in the consequent (for example, the crisp values for a 0-order Sugeno reasoner describing what “high protein concentration” means) are not properly set, under- or overestimating the outcome of the model. In this case, unless some automatic calibration is used, some trial-and-error should be helpful to determine the values able to reproduce the experimental data.

Finally, the last possibility is that there is a gap in the available domain knowledge, which mandates further investigation and additional laboratory experiments before going back to in silico activity.

## Tip 8: Check that the sum of rules firing is not 0 (for example, using ANDs)

One of the key step for fuzzy inference is obtaining the membership values of your input to the defined fuzzy sets. If an input falls in an area of the universe of discourse that is not covered by a fuzzy sets, this value will be equal to 0. Analogously, when defining complex fuzzy rules with logic operators (for example, AND), it can happen that the application of such operators results in a degree of satisfaction of the rule equal to 0. When this happens, the result of the inference can be mathematically undefined (usually because of a division by 0). We suggest to define fuzzy sets on all the universe of discourse or restrict the latter to avoid such situation. Moreover, be aware of how the software that you are using to implement your fuzzy model handles this kind of exceptions, in order to be able to immediately pinpoint and solve the problem when it shows up. Software implementations generally display some kind of warning or error, when they fail to perform an arithmetic operation.

## Tip 9: Use only open source programming languages and software programs

When you start a new fuzzy logic project, you might have the chance to choose which programming language and software to use. This critical decision is often underestimated in universities and research centres. Without any doubts, we definitely recommend you to choose an open source programming language (such as Python, R, and GNU Octave) and to avoid proprietary software.

Using open license programming languages would bring several benefits to your scientific project. You would be able to openly share your code files among your collaborators, without worrying about who bought the right license and who did not. Moreover, if you changed institution or job or company, you would be able to bring your scripts and code files with you and to work with them again in your new work environment. Your software, if shared, would allow the reproducibility of your analyses and the double-check of the results you obtained, making your study more robust. This aspect is particularly important when dealing with data of patients [28].

On the other hand, using proprietary software would bring several drawbacks, including the impossibility to share your code with collaborators who do not have the license for that software. Additionally, since many universities and research centers worldwide are public and funded with taxpayers’ money, we believe that it would be unfair to use these funds to buy proprietary software, while better alternatives are available.

Out of all the programming languages available, we recommend you to choose Python, which is both the most used programming language in the world [29] and the most popular programming language employed in data science and machine learning worldwide [30]. In Python, we recommend the usage of the Simpful [15] and pyFUME [31] software libraries.

R is the most employed programming language in bioinformatics, especially because of the Bioconductor platform [32]. If you can program in R, we suggest you to use the FuzzyR software package [33]. For scientific visualization, we recommend the ggplot2 [34] package in R and the matplotlib [35] software library in Python.

If you feel more familiar with GNU Octave, we endorse the usage of the GNU Octave fuzzy logic toolkit [36,37].

The same goes with technology: if you buy a new computer, install an open source operating system (OS), such as Linux Ubuntu [38]. Following the same school of thought, if you have the chance to start a new distributed computing system from scratch, build it on an open source cluster engine such as Apache Spark [39]. For document handling, we advise using LibreOffice [40] and so on. Just avoid proprietary products.

Moreover, we suggest to publish your software code on online repositories under open license such as GitHub, GitLab, and SourceForge. This spread of the code would allow other users online to reuse your software code and to make your analyses more reproducible. If you are authorized, we also recommend you to share your dataset online on open public datasets such as FigShare [41], Zenodo [42], University of California Irvine Machine Learning Repository [43], Kaggle [44], or even on your own data repository [45]. This way, other researchers worwlide would be able to reanalyze your data and discover something new and meaningful about them or event to spot some mistakes that would have led to false discoveries [46]. When the data are publicly shared, a scientific study can have a higher impact in terms of citations [47].

Finally, if you happen to have a say in the scientific journal where to submit your article, we recommend you to pick an open access one. Open access journals, in fact, allow anyone in the world, including people from low-income countries, to read a scientific article and to benefit from its discoveries. Of course, open access articles get more citations too [48], which mean more visibility for your study and your effort. One can find lists of open access journals on bioinformatics and on health informatics on ScimagoJR [49,50].

## Tip 10: Document everything, especially how you designed the model

One of the most important component of a successful scientific project, not only in fuzzy logic, is the documentation writing. Having a document containing a clear, detailed, specific documentation on all the details of your scientific project and on how you made all its decision is a key asset: we therefore recommend you to write precise, detailed documentation about any aspect of your fuzzy logic project, following already-published guidelines on documentation [51–53].

In your documentation, describe each variable, statuses, interactions, and sets of your fuzzy logic model, and why you arranged them in the way you arranged. Describe all the files you employed. Describe all the software packages, their functions you use, and why you chose them all. Describe the dataset through metadata [54]; follow the example of Galileo Galilei [55].

This documentation will be an invaluable asset and treasure for anyone who would like to study your project and for your future self as well. A well-written documentation, moreover, can be the foundation of a paper draft, allowing the scientific writing phase to happen more quickly and efficiently. You can also consider using your documentation to release a tutorial on your analysis, as done by Melody K. Morris and Thomas Cokelaer for their CNORfuzzy software package [56,57] on Bioconductor, for example.

## Conclusions

Describing a biomedical scenario or environment through a statistical model is a common practice in scientific research, and fuzzy logic can be an useful tool for this scope, especially because it can handle and represent uncertainty. However, it is possible to make mistakes while designing fuzzy logic models, especially when done by beginners and students without a deep knowledge on fuzzy logic. In this study, we describe a guideline on what to do and what not to do when developing a fuzzy logic model to represent a biological or medical system, in an easy way, using simple words that anyone can understand.

To the best of our knowledge, no other study reports guidelines or best practices on fuzzy logic modeling in biomedical informatics exists in the scientific literature at the moment. We fill this gap by presenting these quick tips that, if taken into practice, can allow researchers and students to develop better fuzzy logic models and ultimately obtain more robust and valid results and outcomes.

One of the advantages of fuzzy models lies in their interpretability and openess to inspection. Because of this, it is important to try to keep your model as simple as possible and, at the same time, involve experts in the field both in the definition of the model and in the interpretation of its outputs. Thanks to the use linguistic terms and a formalism close to human language, even nonexperts in fuzzy logic might find patterns or discover new insights by inspecting the outputs of your model.
